# The measure and significance of Bateman's principles

**DOI:** 10.1098/rspb.2013.2973

**Published:** 2014-05-07

**Authors:** Julie M. Collet, Rebecca F. Dean, Kirsty Worley, David S. Richardson, Tommaso Pizzari

**Affiliations:** 1 Department of Zoology, Edward Grey Institute, University of Oxford, Oxford OX1 3PS, UK; 2 School of Biological Sciences, University of Queensland, Brisbane, Queensland 4072, Australia; 3 Department of Genetics, Evolution and Environment, University College London, The Darwin Building, Gower Street, London WC1E 6BT, UK; 4 Department of Evolutionary Biology, Uppsala University, Norbyvägen 18D, Uppsala 752 36, Sweden; 5 Centre for Ecology, Evolution and Conservation, School of Biological Sciences, University of East Anglia, Norwich Research Park, Norwich NR4 7TJ, UK

**Keywords:** Bateman principles, sexual selection, sex roles, sexual dimorphism, polyandry, *Gallus gallus*

## Abstract

Bateman's principles explain sex roles and sexual dimorphism through sex-specific variance in mating success, reproductive success and their relationships within sexes (Bateman gradients). Empirical tests of these principles, however, have come under intense scrutiny. Here, we experimentally show that in replicate groups of red junglefowl, *Gallus gallus*, mating and reproductive successes were more variable in males than in females, resulting in a steeper male Bateman gradient, consistent with Bateman's principles. However, we use novel quantitative techniques to reveal that current methods typically overestimate Bateman's principles because they (i) infer mating success indirectly from offspring parentage, and thus miss matings that fail to result in fertilization, and (ii) measure Bateman gradients through the univariate regression of reproductive over mating success, without considering the substantial influence of other components of male reproductive success, namely female fecundity and paternity share. We also find a significant female Bateman gradient but show that this likely emerges as spurious consequences of male preference for fecund females, emphasizing the need for experimental approaches to establish the causal relationship between reproductive and mating success. While providing qualitative support for Bateman's principles, our study demonstrates how current approaches can generate a misleading view of sex differences and roles.

## 1. Introduction

In a pioneering study published in 1948, Bateman [1] extrapolated from experimental results in *Drosophila melanogaster* to propose that intrasexual selection is normally more intense in males because typically (i) compared with females, males have higher variance in number of mates (i.e. mating success); (ii) males also have higher individual variation in the number of offspring produced (i.e. reproductive success) than females and (iii) the slope of the relationship between mating and reproductive success (Bateman gradient) is steeper in males than in females. These observations became known as Bateman's principles [2,3]; their formalization through selection analysis [4,5] marked the advent of modern sexual selection theory [5–7]. Bateman's principles provide a conceptual explanation for Darwin's observations that sexual selection is typically more intense in males and that males are often ‘eager’ to mate whereas females are ‘coy’ [8]. This Darwin–Bateman paradigm represents the foundation of our understanding of the evolutionary ecology of sex-specific selection, sex roles and sexual dimorphism [3,9].

A number of empirical studies have provided qualitative support for Bateman's principles and their link with sex roles [3,10–17]. Recent work, however, has highlighted a number of problems with both the measurement and interpretation of Bateman's principles [18–21], calling into question the magnitude of the estimated sex differences and their eco-evolutionary significance for four main reasons. First, Bateman gradients are measured by inferring mating success from offspring parentage [5,19,22,23], based on the assumption that an individual sires offspring with each of its mates. However, this assumption is problematic especially when polyandry generates sperm competition, which can result in males gaining no paternity despite behaviourally successful matings. Inferring mating success from offspring parentage in the absence of independent data on mating behaviour can thus introduce a source of error in estimates of male Bateman gradients. In most studies, measures of mating success based on parentage also capture, implicitly or explicitly, components of offspring survival (e.g. [24], reviewed in reference [25]), which can further bias estimates of Bateman gradients [26,27]. However, the magnitude of this potential source of error remains untested. Moreover, because—all else being equal—the probability of a male fertilizing at least one egg is a function of clutch size, the bias introduced by inferring male mating success from parentage is expected to be inversely proportional to average clutch size [22]. A better alternative to inferring mating success from offspring parentage is to observe mating behaviour directly and record mating rates for each individual [14]. However, the use of mating rates often fails to distinguish between an individual mating multiple times with the same partner, or mating once with multiple partners [28]. This distinction is crucial, because remating with the same partner and mating with multiple partners may influence the reproductive success of an individual in drastically different ways. In males, for example, remating with the same female would influence a male's chances of fertilizing her eggs, whereas mating with multiple females would directly contribute to his mating success. One would therefore need to measure the actual number of mates for each individual, controlling for the potentially confounding effect of number of matings obtained with each mate. This requires intensive observations of individually tagged males and females [17,29].

Second, measuring the Bateman gradient as the slope of the linear least-square regression of reproductive success over mating success may be simplistic, because even with unlimited availability of mating opportunities the reproductive success of an individual is, at some point, limited by intrinsic reproductive costs such as the production of enough gametes [30]. It is therefore plausible that variation in reproductive success may be better characterized as a positive quadratic function of mating success. This is important because it suggests that sex differences may occur not only in the slope of linear functions of reproductive success (mating success), but also in the shape of such functions. The comparison between the maximum of the curvilinear function with the average mating success of a population enables one to test whether the reproductive success of members of one sex in a population is limited by the availability of mating opportunities (maximum > average mating success) or by intrinsic reproductive costs (maximum < average mating success [30]).

Third, polyandry makes the relationship between mating and reproductive successes more complex [9,31–33]. With polyandry, the reproductive success of an individual male is the product of his mating success, the fecundity of his mates (mate fecundity) and the proportion of ova fertilized within the clutch of each female he mated with (paternity share). Because Bateman gradients are measured as the slope of the univariate least-square regression of reproductive success over mating success, they capture both direct selection on mating success, but also indirect selection on other components of reproductive success if mating success covaries with mate fecundity and/or paternity share. Hence, the Bateman gradient behaves as a selection differential rather than act as a selection gradient as it encompasses direct and indirect selection on mating success [34]. For example, the male Bateman gradient can be inflated via paternity share if, for a given mate fecundity, males that mate with more females have on average a higher paternity share of each clutch [35]. Alternatively, by mating with more females, males might suffer from reduced paternity share through trade-off mechanisms [36]. One way to measure the relationship between mating and reproductive successes controlling for these covariances is to use a traditional multivariate approach such as1.1
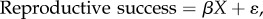
where *β* is the vector of partial regression coefficients on mating success, mate fecundity and paternity share, *X* the matrix of phenotypic values for those traits and *ɛ* an error term of 0 mean [34].

Finally, it is becoming clear that females can also have positive Bateman gradients, even in species with typical ‘sex roles’ [18,37–39], which suggests that sexual selection for mating success might also be significant in females [2,18,40–43] potentially owing to cumulative benefits associated with mating [44,45]. Note that this is not necessarily inconsistent with the Darwin–Bateman paradigm provided that the Bateman gradient is steeper in males than in females in a given species. However, the significance of female Bateman gradients and the relative strength of sexual selection on female mating success remain debated [22,45,46]. Alternatively, a positive female Bateman gradient may arise if more fecund females are exposed to higher mating rates solely because they are more attractive to males, thus reversing the causality of the relationship between female mating and reproductive successes [22,47,48]. Explicit tests of the significance of female Bateman gradients remain scarce.

Here, we combine an experimental approach with multivariate analyses to address these issues and resolve the significance of Bateman's principles in replicate groups of red junglefowl, *Gallus gallus*. First, we test Bateman's principles using the traditional approach of inferring mating success from genetic parentage of offspring (MS_gen_) and univariate regressions of reproductive success on mating success inferred from offspring parentage. Second, we measure mating success using fine-grained mating behaviour data to measure the bias introduced by the traditional use of mating success inferred from parentage. Third, we use a multivariate approach to quantify the relationship between male mating success and reproductive success, controlling for mate fecundity, paternity share and their covariances with mating success. Finally, we explore the causality of female Bateman gradients by testing whether variation in female mating success causes changes in female reproductive success.

## 2. Material and methods

### (a) Observations and parentage assignment in semi-natural conditions

We studied a population of red junglefowl at the field station of the University of Oxford between May–September 2007 and August–September 2008, as detailed in reference [35]. Briefly, we observed 13 replicate groups of three adult males and four adult females in outdoor pens, typical group size and sex ratio in natural groups [49]. In each replicate group, we monitored mating behaviour for 10 consecutive days. We collected all the eggs laid in each group from the second day of observation (i.e. the first day in which inseminations may have resulted in fertilization) and for the subsequent 10 days (i.e. day 2–11 inclusive, see the electronic supplementary material, table S1 on variation in female reproductive success). One group departed from this pattern as egg collection occurred from day 7 to day 16. We tested the effect of removing this group from the analysis, but as no qualitative difference was observed, we present only the results of the full dataset. We incubated eggs artificially for 7 days when we collected the embryos of fertilized eggs. In order to measure whether sperm depletion was likely to create sexual selection in females, we recorded the percentage of eggs that showed no evidence of embryo development (indicating that the eggs were either not fertilized or suffered embryo death within the first hours of development) in five of the 13 replicate groups. In these five groups, 11% of the eggs showed no sign of embryo development. More than half of these eggs were laid in the first days of egg collection, suggesting that they were not fertilized owing to the expected delay between insemination and fertilization [50]. Excluding eggs laid in the first 2 days, reduced the proportion of eggs with no sign of embryo development to 5%. Collectively, these results indicate that if some fertilizations went undetected in the study, then these represented a small proportion of the eggs. All embryos (*n* = 254) were genotyped at seven variable microsatellite loci [50], their paternity and maternity were successfully assigned in Cervus 3.0 ([35,51,52] and the electronic supplementary material, table S2).

### (b) General analytical approach

Reproductive success in males and females was calculated as the number of embryos produced by each individual. Mating success inferred from genetic parentage of the offspring (MS_gen_) was calculated as the total number of mates with whom an individual produced offspring. Importantly, we also measured ‘total’ male and female mating success (MS_tot_) by adding to MS_gen_ any additional mate with whom a focal individual had been observed successfully copulating during the 10 day period of observation but to which no offspring were assigned. To control for the potential effects of mating repeatedly with the same mate, we entered as a covariate the average number of successful matings that a focal individual was observed to have with each mate. As no difference was observed by adding this covariate, we did not report results of analyses that included it. In addition, for males, mate fecundity was calculated as the average number of eggs laid by all females with whom a male produced offspring, corrected by the number of eggs laid by all females with whom a male was observed to copulate successfully (see calculation of MS_tot_ above). Accordingly, the paternity share of a male was calculated as the proportion of embryos sired out of the total number of embryos produced by all the females with whom he successfully mated. We calculated the opportunity for total sexual selection *I* (*I*_T_ sensu [35]) as standardized variance in reproductive success [4,53,54]:2.1
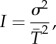
where *σ*^2^ represents the variance in reproductive success, and 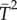 is the square mean of the reproductive success of members of one sex in a group. Similarly, we calculated the opportunity for sexual selection on mating success, *I*_S_ (*I*_M_ sensu [35]) as the standardized variance in mating success2.2
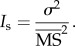
We also used a novel method described by Moorad & Wade [55] to determine the proportion of *I* explained by the different components of male sexual fitness, namely mating success, mate fecundity and paternity share. In brief, we partitioned *I* into additive components (either only *I*_S_ in the univariate analysis, or with the other components of male sexual fitness in the multivariate analysis), and significance was tested by normal bootstrapping [56] to estimate confidence intervals. Finally, to obtain results comparable with other organisms with various population and clutch sizes, we produced a standardized measure of Bateman gradients [2]. We standardized Bateman gradients as usually done for selection gradients by dividing reproductive success by the average population score (relative fitness), whereas each individual trait (mating success, mate fecundity and paternity share) was subtracted by its population mean and then divided by its standard deviation in the population, to obtain a mean of zero and a standard deviation of unity.

In the models comparing different ways of calculating the Bateman gradients, estimates of all gradients were deduced from a simple linear model of male or female reproductive success and different predictors [5]. To obtain the significance of fixed effects and overall fit of models, we performed generalized-mixed models (lmer, library lme4 in R v. 2.15.2, R Core Team [57]), with replicate group as random effect with 13 levels. Some birds were used in more than one replicate group (four males used in two groups, four in three groups, one in four groups, nine females in two groups and seven females in three groups). Repeatability in the components of reproductive success (total reproductive success, mating success, mate fecundity and paternity share) across groups was consistently low in males [35]. For males used in multiple groups within the same reproductive season, relative male's reproductive performances in one group were poor predictors of their relative performance in the next group (reproductive success: adj. *R*^2^ = −0.07; mating success: adj. *R*^2^ = −0.07; mate fecundity: adj. *R*^2^ = −0.08; paternity share: adj. *R*^2^ = −0.08). Similarly, the relative reproductive and mating success of a female in a group did not predict her reproductive success and mating success in the next replicate group within the same year (reproductive success: adj. *R*^2^ = −0.01; mating success: adj. *R*^2^ = −0.05), suggesting that the reproductive performance of these birds was largely contingent on the dynamics of different replicate groups rather than consistently determined by inherent properties of the individual or by seasonal patterns. Nevertheless, bird identity nested within group was fitted as an observation-level random effect to control for pseudo-replication and overdispersion. In all mixed models, the response was male or female reproductive success which conformed to a Poisson distribution; hence, we used a log link function to test the fixed effects. The significance of each fixed effect was tested by a log-likelihood ratio test comparing models fitted by the Laplace approximation including or excluding the tested fixed effect. However, because the log-likelihood ratio test is considered non-conservative for fixed effects [58], and because the log link function does not directly test the linear relationship that the Bateman gradient aims to describe, we calculated confidence intervals for each estimate of slope with bootstrapping methods. We first fitted a linear-mixed model (lme, library nlme, in R v. 2.15.2) on reproductive success with the fixed effects. We then shuffled the residuals given by this model, added them to the fitted values and refitted the model to these newly created dataset [59]. To be conservative, we repeated this bootstrapping method 2000 times on mixed models including either (i) the bird identity, (ii) the group of birds and (iii) the bird identity nested within the group of birds as random effects. The results presented include 0.95 confidence intervals given by these simulations.

#### (i) The traditional measure of Bateman's principles

We calculated the total opportunity for selection (*I*), and the opportunity for sexual selection (*I*_S_) for males and females as the standardized variance in mating success inferred from genetic parentage (MS_gen_). We also calculated confidence intervals on *I* indices and the percentage of *I* explained by *I*_S_ [55]. Male and female Bateman gradients were calculated independently by using male and female MS_gen_ as fixed effects in the models above. To compare male and female Bateman gradients, we merged male and female datasets and computed mixed models fitted with a Poisson distribution that used, without intercept (i) sex and MS_gen_ as fixed effects and (ii) sex, MS_gen_ and the interaction between both as fixed effects. The comparison between (i) and (ii) by a log-likelihood ratio test quantified the statistical significance of the difference between male and female Bateman gradients. We also tested for quadratic effects by adding 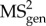 as a fixed effect and calculated confidence intervals for MS_gen_ and 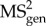 as previously described.

#### (ii) The bias introduced by inferring mating success from offspring parentage

To investigate the effect of matings not resulting in fertilization on Bateman's principles, we recalculated male and female *I*, *I*_S_ and Bateman gradients as above, using MS_tot_ instead of MS_gen_. We then compared the general fit and the Akaike information criterion (AIC) of models based on MS_gen_ versus MS_tot_.

#### (iii) The multivariate measure of Bateman's principles

To test whether the opportunity for sexual selection (*I*_S_) and the Bateman gradients were artificially inflated by the covariances of mating success with other components of male reproductive success [35], we added male mate fecundity and paternity share to the model. We first calculated opportunity of selection on mate fecundity and paternity share as described in reference [55]. Second, we tested the effect of adding mate fecundity and paternity share in the calculation of the Bateman gradient by comparing the goodness of fit and the AIC of the generalized-mixed models. We also tested the effect on male reproductive success of the covariances between all components of male reproductive success by adding second-degree interactions to the model, but as this was not significant we do not report it here.

#### (iv) The significance of female Bateman gradients

We explored the hypothesis that a positive female Bateman gradient arises because mating with additional males causes an increment in female fecundity (adaptive hypothesis). The adaptive hypothesis predicts a specific temporal pattern, whereby female reproductive rate (i.e. the probability to lay a fertile egg on a given day) increases over successive days for polyandrous females, as they accumulate additional partners, but remains constantly low in monandrous females. There are two alternative (null) hypotheses for a positive female Bateman gradient. First, inherently more fecund females mate with more males. Second, even if all females mate with the same number of males, then inherently more fecund females will, in principle, by producing more eggs, have a higher probability of producing offspring sired by more males. However, measuring mating success based on behavioural data should make this latter explanation less likely to apply to our study. Neither of these alternative scenarios predicts temporal changes in the reproductive rate of polyandrous versus monandrous females over the course of a trial. We therefore analysed variation in the probability that a female would lay a fertile egg on a given day over successive days of the trial in relation to her cumulative mating success (female MS_tot_ = 1, 2 or 3), with a mixed model fitting a binomial distribution and, including trial day, MS_tot_, and day × MS_tot_ interaction as fixed effects.

We then conducted an experiment to test the causal relationship between female mating and reproductive successes. Monoandry and polyandry treatments were sequentially conducted in random order on 13 females. Females received controlled matings [60] in the afternoon on 3 consecutive days, either three times with the same male (monoandry) or with three different males (polyandry). Egg laying rate was monitored for each of the females for the 7 days following the treatment [50]. Females were housed in groups, and eggs were assigned to females using orally administered coloured lipid dyes [60]. We compared the number of eggs laid by a female following the polyandry treatment with that of the same female following the monoandry treatment, with a paired *t*-test. As our sample was rather limited (*n* = 13), we conducted a power analysis to estimate the likelihood of finding an effect size similar to the one observed in the semi-natural groups. We ran a model simulating a Poisson distribution with the same sample size and average number of eggs laid as in the controlled mating experiment. The power analysis showed that we had 79.2% chance of detecting the difference in fecundity between females mated to one and females mated to two males observed in the semi-natural groups. Note that this power analysis is conservative because it does not consider the within-female paired design.

## 3. Results

### (a) The traditional measure of Bateman's principles

**Figure 1.**
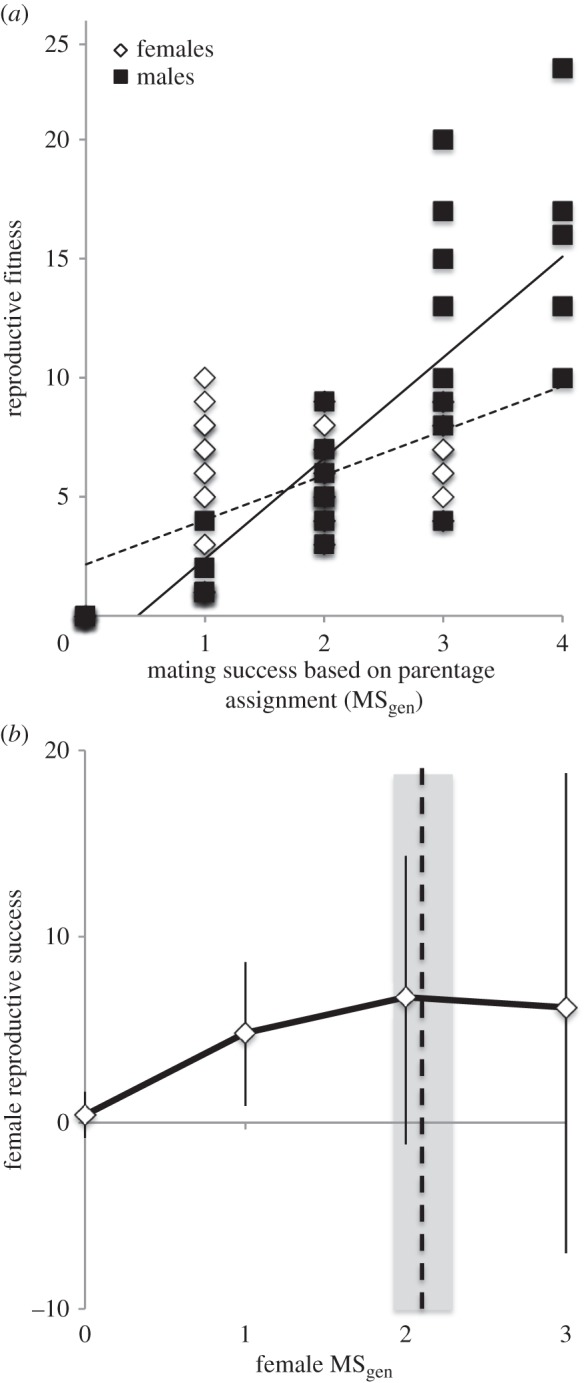
(*a*) Male (solid squares and line) and female (empty diamonds, dashed line) Bateman gradients based on MS_gen_. (*b*) The quadratic relationship between female MS_gen_ and reproductive success over a MS_gen_ range of 0–3, vertical bars represent CI. The function shows a maximum for MS_gen_ = 2. The vertical dashed line shows (approximately) mean female MS_gen_ across groups, the shaded section around the line represents SE around the mean (2.13 ± 0.16).

**Figure 2.**
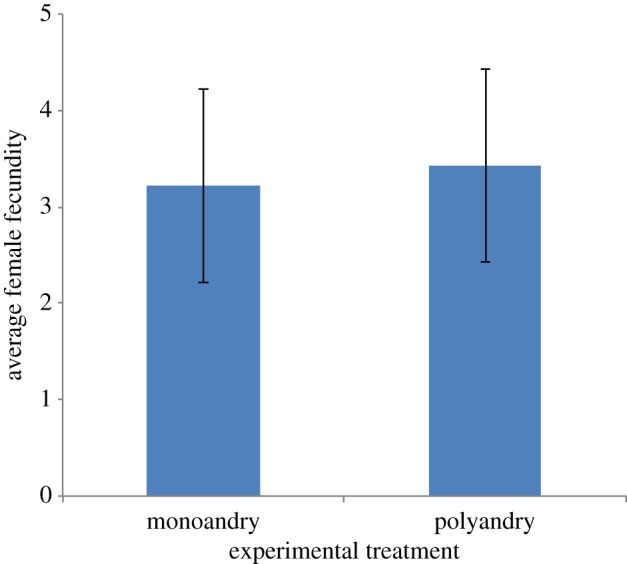
Average number of eggs produced by females mated three times with a single male (monoandry) or once with each of three males (polyandry). (Online version in colour.)

The opportunity for selection was almost twice as high in males as in females (table 1). Similarly, the opportunity for sexual selection on mating success was approximately four times higher in males than in females, and was significantly positive in both sexes (table 1). The variance in mating success explained 56.9% of the variance in reproductive success in males, but only 24.1% in females (table 1). Finally, the male Bateman gradient was significantly steeper than the female gradient, as indicated by the comparison between a model, including the fixed effects sex and MS_gen_, and a model, including sex, MS_gen_ and the interaction between both (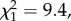 *p* = 0.002; figure 1*a*). However, the Bateman gradient was significantly positive in both males and females (figure 1*a* and table 1). Standardized measures of male and female gradients produced a qualitatively similar pattern (table 1). We detected a positive quadratic relationship between reproductive and mating successes in females (estimate MS_gen_ = 5.65 [3.60; 7.60], estimate 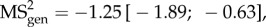 figure 2*b*) but not in males (estimate MS_gen_ = 1.84 [−0.65; 4.32], estimate 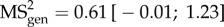). The female quadratic function was maximized when females had 2.26 partners (female MS_gen_ = 2.26 [CI 0.95; 6.04]), which was very close to the average female mating rate in the population (average MS_gen_ = 2.14, figure 1*b*).

**Table 1.** Outcome of different models describing the relationship between reproductive success and mating success in males and females. The opportunity for selection indices, *i*, were obtained following [54], *i* (% of I) is the proportion of the variance in reproductive success explained by a parameter, fit of the model is the proportion of the variance in reproductive success explained by the complete model. Gradients were obtained with a linear model and standardized following [2]. The overestimation of the Bateman gradient calculates how much the Bateman gradient, measured using the traditional univariate approach, overestimates the relationship between reproductive and mating success. We obtained *p* values and AIC through a generalized-mixed model fitted with a Poisson distribution (see methods).

model	sex	parameter	*i* [CI]	*i* (% of *I*)	fit of the model (% of *I*)	gradient [CI]	gradient (standardized)	AIC	overestimation of the Bateman gradient (%)	overestimation on standardized data (%)	*p*-value
traditional	male	mating success	0.45 [0.27; 0.65]	56.9	56.9	4.23 [3.66; 5.21]	0.81	59.9			<0.001
	female	mating success	0.11 [0.00; 0.22]	24.1	24.1	1.87 [1.15; 2.59]	0.34	103.6			<0.001
MS_tot_	male	mating success	0.34 [0.16; 0.53]	42.5	42.5	2.82 [1.73; 3.92]	0.60	81.6	50	35	<0.001
	female	mating success	0.03 [−0.06; 0.09]	5.4	5.4	1.50 [0.44; 2.45]	0.24	116.8	25	42	0.001
multivariate	male	mating success	0.22 [0.08; 0.37]	27.8	96.3	1.67 [0.72; 2.62]	0.35	22.5	153	131	<0.001
		mate fecundity	0.00 [−0.02; 0.02]	0.0		1.79 [1.33; 2.25]	0.42				<0.001
		paternity share	0.55 [0.32; 0.80]	68.5		16.77 [13.70; 19.71]	0.70				<0.001

### (b) The bias introduced by inferring mating success from offspring parentage

Overall, 29.4% of the pairs that were observed mating did not sire offspring together and were therefore missed by estimates of mating success based on genetic parentage assignment (i.e. male and female MS_gen_). When taking into account matings that did not result in fertilization by correcting data on mating success based on parentage with behavioural observations (MS_tot_), the opportunity for sexual selection on mating success dropped in males (male *I*_S_ = 0.34 [CI: 0.16; 0.53]) and became null in females (female *I*_S_ = 0.03 [CI: −0.06; 0.09]; table 1). Similarly, when correcting data on mating success based on parentage with behavioural observations, the variance in mating success explained 42.5% of the variance in male reproductive success and only 5.4% of the variance in female reproductive success (table 1). Correcting data on mating success based on parentage with behavioural observations also greatly changed the models estimating male and female Bateman gradients (table 1). The Bateman gradient remained significantly positive in both males and females (table 1) after correcting data on mating success based on parentage with behavioural observations. However, both male and female gradients were reduced, indicating that the traditional use of mating success based on offspring parentage can lead to considerable errors in the estimates of both unstandardized and standardized Bateman gradients for males and females (table 1).

### (c) The multivariate measure of Bateman's principles

Taking into account the covariances between male components of reproductive success explained more than 96% of the opportunity for selection in males (*I*), resulting in a significant improvement on the explanatory power of male reproductive success and in a lower AIC than that of the univariate model based on MS_tot_ (table 1). This multivariate model identified three independent sources of selection on male reproductive success: mating success (i.e. Bateman gradient), mate fecundity and paternity share (table 1), and revealed that, by failing to control for covariances between these components of male reproductive success, the traditional univariate approach overestimated the male Bateman gradient by more than 153% of the unbiased selection gradient (table 1). Again, the use of standardized gradients qualitatively agreed with this result (table 1).

### (d) The significance of female Bateman gradients

Contrary to the predictions of the adaptive hypothesis and consistent with those of the null hypotheses, we found that the probability of laying an egg did not change with time (i.e. throughout the duration of a trial), but was consistently higher for females with higher mating success (MS_tot_: 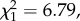 *p* = 0.01, day: 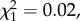 *p* = 0.88, day × MS_tot_: 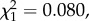 *p* = 0.78).

In the controlled mating experiment, females did not produce more eggs when they were mated to different males (polyandry) than when they were repeatedly mated to the same male (monoandry, *t* = −0.41, d.f. = 12, *p* = 0.69; figure 2).

## 4. Discussion

Bateman's principles are a cornerstone of modern sexual selection theory, yet intense recent debate has called into question their measure and relevance in studying sex differences [18,21,61–64]. Our study contributes to this ongoing debate by demonstrating that the traditional use of MS_gen_ can generate severely misleading estimates of Bateman's principles. Because matings can fail to result in fertilization, particularly in polyandrous species, the number of females with whom a male mates successfully can only be as great as the number of females with whom he sires offspring and typically lower than this (i.e. MS_tot_ ≥ MS_gen_). This inequality means that inferring mating success from offspring parentage is likely to introduce a systematic bias, by overestimating the opportunity of sexual selection on males. Importantly, for a constant share in paternity and mate fecundity, the total reproductive success of a male is directly proportional to his mating success inferred from offspring parentage but is independent from residual mating success that fails to result in paternity. Failing to consider this latter component of mating success is therefore likely to overestimate the steepness of the male Bateman gradient [17], and, because the probability of fertilizing at least one egg increases with clutch size, this bias is likely greater in species with smaller clutches.

Another large source of error arises from the covariance of male mating success with the other constituents of male reproductive success, namely mate fecundity and paternity share, which are not considered in the traditional univariate approach to Bateman's principles. In red junglefowl, male mating success and paternity share may covary for different reasons. In our study population, males with high mating success also tend to enjoy high paternity share. This appears to be mediated by social dominance which allows a male to access more females and repeated mating opportunity with each female, which conveys a sperm competition advantage (high paternity share), likely through a replenishment of the male's sperm in the female's sperm storage tubules [35]. An alternative scenario may arise when males invest in either mating opportunities or fertilization efficiency through alternative mating tactics [65]. Under such conditions, males with high mating success may be poor sperm competitors and suffer low paternity share, whereas males with high paternity share may suffer low mating success, resulting in negative (rather than positive) covariance between mating success and paternity share. Several studies have shown that in domestic fowl, socially dominant males tend to produce ejaculates of lower sperm swimming velocity than the ejaculates produced by their subordinates [66–68]. Controlling for mating frequency and number of sperm inseminated, higher sperm velocity would give subordinate males an advantage in sperm competition [69]. A negative covariance of mating success and paternity share may also be more likely to occur in species where repeated matings are less relevant, such as for example external fertilizers. Covariance between male mating success and mate fecundity may also introduce significant bias, if for example males with high mating success mated preferentially with more fecund females.

Measuring Bateman gradients taking into account male paternity share and mate fecundity is non-trivial and to the best of our knowledge, few studies have attempted this in the past. Fritzsche & Arnqvist [39] have used the covariance between a trait and the residual reproductive success unexplained by the Bateman gradient to measure the overall selection on mate fecundity and paternity share. This approach, however, is simplistic: the potential covariances between mating success and other components of male sexual fitness make reality more complex. We really need to integrate mating success, mate fecundity and paternity share in a multivariate framework that takes into account their covariances in order to accurately quantify sexual selection on mating success (i.e. the Bateman gradient), mate fecundity and paternity share. Few studies have attempted to quantify such covariances. A recent study of the hermaphroditic snail, *Physa acuta*, found significant covariances between different components of male reproductive success [28]. Similarly, studies of passerine birds forming social pair bonds have quantified the covariance between within- and extra-pair components of male reproductive success, demonstrating that such covariance can represent a substantial source of variation in male reproductive success in some populations [70,71], but less so in others [48,72]. In part, differences between these studies are likely to reflect a combination of biological factors, such as population size, patterns of variation in polyandry, mate availability and clutch size, and future studies should seek to resolve how Bateman principles can be modulated by these factors. Some discrepancies may also reflect methodological differences. For example, Pélissié *et al*. [28] attributed variance in remating rates with the same female to sexual selection on male mating success [28], although in red junglefowl mating repeatedly with the same female has been shown to have a direct influence on paternity share [35]. Similarly, the distinction between within- and extra-pair reproductive success is not readily applicable to species that lack social pair bonds.

Our results also confirm the concerns expressed by previous studies over the interpretation of female Bateman gradients [47,48]. Positive female Bateman gradients, while significantly lower than corresponding male Bateman gradients, have been shown in a number of species, for example, bank voles [15], wild turkey [16] and *Drosophila* [14,18]. However, the causality of the relationship between female mating and reproductive successes is often unclear. Using a traditional approach, one would be led to conclude that red junglefowl females are selected to mate with multiple males, and optimize mating success at around two males. Because this value does not differ significantly from the average female mating success observed in these groups, one would also be led to conclude that females are in control of mating rates [30]. However, these conclusions are misleading. Such a positive female Bateman gradient is likely to arise as a spurious consequence of more fecund females mating with more males, rather than as an adaptive consequence of female polyandry. That the adaptive hypothesis is unlikely to explain our observation of a positive female Bateman gradient is indicated by (i) the lack of a time lag, which would be required for polyandry to cause an increment in female fecundity, and (ii) the lack of any evidence that mating with additional males causes an increase in fecundity in the pair-wise controlled experiment. The null hypothesis, that females that are inherently more fecund attract more mates, is entirely consistent with our results and also more plausible given the biology of the study species. This pattern may arise through both female- and male-driven mechanisms. Female fowl tend to display a higher propensity to mate in periods when they ovulate than in periods when they do not, and males have been shown to preferentially target ovulating females [73], indicating that females that lay more eggs are likely to be more sexually promiscuous. Kokko *et al*. [62] emphasize the importance of establishing causality to understand Bateman's principles. Our study indicates that this is particularly important when it comes to the interpretation of female Bateman's gradients.

Clearly, the approach that we adopted to measure male reproductive success also suffers from its own limitations. First, we may have missed a proportion of matings. Although we monitored the populations throughout the daily peaks in mating activity (early morning and late afternoon [74]), it is certainly possible that some mating activity went unnoticed. If these matings resulted in fertilization, then they would have been accounted for by our measure of mating success which complements behavioural data with cases in which paternity data identifies mating events undetected by behavioural observations. In actual fact, such cases were rare in our study (7.3% of the total number of mates was missed by behavioural data and detected through offspring parentage assignment). This does not eliminate the possibility that we may have missed mating events that failed to result in fertilization. However, undetected matings that failed to result in fertilization would mean that the discrepancy between MS_gen_ and MS_tot_ is even greater than reported by our study, and thus the bias introduced by inferring mating success exclusively from offspring parentage is consequently also even greater than our study estimates. Second, the use of behavioural data requires some careful consideration and may need to be tailored to the specifics of different study organisms. In our study, we considered only sexually mature males and females during their breeding season, and counted only mating events that comprised the entire succession of behavioural steps concluding with a successful cloacal contact [35]. A more careful approach may be required by studies of natural populations with limited possibilities for experimental control, less complete information on the reproductive status of different individuals or where matings observed represent a non-random subset of the mating occurring in the population [75]. In addition, although we incubated eggs artificially and sampled embryos early in their incubation (on day 7 of 21 day incubation period), we cannot entirely rule out the risk that our measure of reproductive success may have been influenced by embryo mortality at very early stage of development (i.e. within the first approx. 24 h from fertilization), which may be difficult to detect without molecular assays. However, the proportion of eggs that was deemed infertile in our study represented approximately 5% of all the eggs collected. Therefore, if it occurred, undetected early embryo mortality would appear to have had only a modest contribution.

Finally, our study shows that while efforts to reduce error can generate estimates of Bateman's principles that are quantitatively drastically different from estimates derived from traditional approaches, the patterns of sex-specific differences observed are qualitatively entirely consistent with the three Bateman's principles. Namely, we found higher *I* and *I*_S_ in males than in females and stronger sexual selection on male rather than on female mating success. Therefore, while our study suggests that previous estimates of Bateman's principles are likely biased, it also provides a robust confirmation that these principles are real and not entirely artefacts of methodological limitations. In conclusion, our experiment confirms the validity of Bateman's principles in a semi-natural population, but simultaneously reveals the necessity to rethink traditional approaches to study the evolutionary ecology of sex roles.

## References

[1] Bateman AJ 1948 Intra-sexual selection in *Drosophila*. Heredity 2, 349–368 (doi:10.1038/hdy.1948.21) 18103134 10.1038/hdy.1948.21

[2] Arnold SJ 1994 Bateman's principles and the measurement of sexual selection in plants and animals. Am. Nat. 144, S126–S149 (doi:10.1086/285656)

[3] Jones AG Rosenqvist G Berglund A Avise JC 2005 The measurement of sexual selection using Bateman's principles: an experimental test in the sex-role-reversed pipefish *Syngnathus typhle*. Integr. Comp. Biol. 45, 874–884 (doi:10.1093/icb/45.5.874) 21676838 10.1093/icb/45.5.874

[4] Crow JF 1958 Some possibilities for measuring selection intensities in man. Hum. Biol. 60, 1–13 13513111

[5] Arnold SJ Duvall D 1994 Animal mating systems: a synthesis based on selection theory. Am. Nat. 143, 317–348 (doi:10.1086/285606)

[6] Shuster SM Wade MJ 2003 Mating systems and strategies. Princeton, NJ, Oxford: Princeton University Press

[7] Jones AG 2009 On the opportunity for sexual selection, the Bateman gradient and the maximum intensity of sexual selection. Evolution 63, 1673–1684 (doi:10.1111/j.1558-5646.2009.00664.x) 19228185 10.1111/j.1558-5646.2009.00664.x

[8] Darwin C 1871 The descent of man and selection in relation to sex. London, UK: J. Murray

[9] Parker GA Birkhead TR 2013 Polyandry: the history of a revolution. Phil. Trans. R. Soc. B 368, 20120335 (doi:10.1098/rstb.2012.0335) 23339245 10.1098/rstb.2012.0335 PMC3576588

[10] Jones AG Rosenqvist G Berglund A Arnold SJ Avise JC 2000 The Bateman gradient and the cause of sexual selection in a sex-role-reversed pipefish. Proc. R. Soc. Lond. B 267, 677–680 (doi:10.1098/rspb.2000.1055) 10.1098/rspb.2000.1055 PMC1690589 10821612

[11] Jones AG Arguello JR Arnold SJ 2002 Validation of Bateman's principles: a genetic study of sexual selection and mating patterns in the rough-skinned newt. Proc. R. Soc. Lond. B 269, 2533–2539 (doi:10.1098/rspb.2002.2177) 10.1098/rspb.2002.2177 PMC1691187 12573067

[12] Jones AG Arguello JR Arnold SJ 2004 Molecular parentage analysis in experimental newt populations: the response of mating system measures to variation in the operational sex ratio. Am. Nat. 164, 444–456 (doi:10.1086/423826) 15459877 10.1086/423826

[13] Andrade MCB Kasumovic MM 2005 Terminal investment strategies and male mate choice: extreme tests of Bateman. Integr. Comp. Biol. 45, 838–847 (doi:10.1093/icb/45.5.838) 21676835 10.1093/icb/45.5.838

[14] Bjork A Pitnick S 2006 Intensity of sexual selection along the anisogamy–isogamy continuum. Nature 441, 742–747 (doi:10.1038/nature04683) 16760976 10.1038/nature04683

[15] Mills SC Grapputo A Koskela E Mappes T 2007 Quantitative measure of sexual selection with respect to the operational sex ratio: a comparison of selection indices. Proc. R. Soc. B 274, 143–150 (doi:10.1098/rspb.2006.3639) 10.1098/rspb.2006.3639 PMC1679881 17134998

[16] Krakauer AH 2008 Sexual selection and the genetic mating system of wild turkeys. The Condor 110, 1–12 (doi:10.1525/cond.2008.110.1.1)

[17] Pélissié B Jarne P David P 2012 Sexual selection without sexual dimorphism: Bateman gradients in a simultaneously hermaphrodite. Evolution 66, 66–81 (doi:10.1111/j.1558-5646.2011.01442.x) 22220865 10.1111/j.1558-5646.2011.01442.x

[18] Snyder BF Gowaty PA 2007 A reappraisal of Bateman's classic study of intrasexual selection. Evolution 61, 2457–2468 (doi:10.1111/j.1558-5646.2007.00212.x) 17725639 10.1111/j.1558-5646.2007.00212.x

[19] Gowaty PA Kim Y-K Anderson WW 2012 No evidence of sexual selection in a repetition of Bateman's classic study of *Drosophila melanogaster*. Proc. Natl Acad. Sci. USA 109, 11 740–11 745 (doi:10.1073/pnas.1207851109) 10.1073/pnas.1207851109 PMC3406809 22689966

[20] Ah-King M 2011 Female sexual selection in light of the Darwin–Bateman paradigm. Behav. Ecol. 22, 1142–1143 (doi:10.1093/beheco/arr109)

[21] Gowaty PA Kim YK Anderson WW 2013 Mendel's law reveals fatal flaws in Bateman's 1948 study of mating and fitness. Fly 7, 28–38 (doi:10.4161/fly.23505) 23360967 10.4161/fly.23505 PMC3660748

[22] Parker PG Tang-Martinez Z 2005 Bateman gradients in field and laboratory studies: a cautionary tale. Integr. Comp. Biol. 45, 895–902 (doi:10.1093/icb/45.5.895) 21676840 10.1093/icb/45.5.895

[23] Uller T Olsson M 2008 Multiple paternity in reptiles: patterns and processes. Mol. Ecol. 17, 2566–2580 (doi:10.1111/j.1365-294X.2008.03772.x) 18452517 10.1111/j.1365-294X.2008.03772.x

[24] Byers J Dunn S 2012 Bateman in nature: predation on offspring reduces the potential for sexual selection. Science 338, 802–804 (doi:10.1126/science.1224660) 23139332 10.1126/science.1224660

[25] Garcia-Gonzáles F 2008 Male genetic quality and the inequality between paternity success and fertilization success: consequences for studies of sperm competition and the evolution of polyandry. Evolution 62, 1653–1665 (doi:10.1111/j.1558-5646.2008.00362.x) 18315573 10.1111/j.1558-5646.2008.00362.x

[26] Arnqvist G 2013 Comment on ‘Bateman in nature: predation on offspring reduces the potential for sexual selection’. Science 340, 549 (doi:10.1126/science.1233413) 23641095 10.1126/science.1233413

[27] Ramm SA Jonker RM Reinhold K Székely T Trillmich F Schmoll T Schielzeth H Freckleton RP 2013 Comment on ‘Bateman in nature: predation on offspring reduces the potential for sexual selection’. Science 340, 549 (doi:10.1126/science.1233298) 23641094 10.1126/science.1233298

[28] Pélissié B Jarne P Sarda V David P In press. Disentangling precopulatory and postcopulatory sexual selection in polyandrous species. Evolution (doi:10.1111/evo.12353) 10.1111/evo.12353 24410424

[29] Anthes N 2010 Bateman gradients in hermaphrodites: an extended approach to quantify sexual selection. Am. Nat. 176, 249–263 (doi:10.1086/655218) 20636132 10.1086/655218

[30] Jones AG Ratterman NL 2009 Mate choice and sexual selection: what have we learned since Darwin? Proc. Natl Acad. Sci. USA 106, 10 001–10 008 (doi:10.1073/pnas.0901129106) 19528643 10.1073/pnas.0901129106 PMC2702796

[31] Simmons LW Parker GA 1996 Parental investment and the control of sexual selection: can sperm competition affect the direction of sexual competition? Proc. R. Soc. Lond. B 263, 515–519 (doi:10.1098/rspb.1996.0078)

[32] Lorch PD 2002 Understanding reversals in the relative strength of sexual selection on males and females: a role for sperm competition? Am. Nat. 159, 645–657 (doi:10.1086/339992) 18707387 10.1086/339992

[33] Wade MJ Shuster SM 2005 Don't throw Bateman out with the bathwater! Integr. Comp. Biol. 45, 945–951 (doi:10.1093/icb/45.5.945) 21676845 10.1093/icb/45.5.945

[34] Lande R Arnold SJ 1983 The measurement of selection on correlated characters. Evolution 37, 1210–1226 (doi:10.2307/2408842) 10.1111/j.1558-5646.1983.tb00236.x 28556011

[35] Collet J Richardson DS Worley K Pizzari T 2012 Sexual selection and the differential effect of polyandry. Proc. Natl Acad. Sci. USA 109, 8641–8645 (doi:10.1073/pnas.1200219109) 22592795 10.1073/pnas.1200219109 PMC3365207

[36] Warner RR Shapiro DY Marcanato A Petersen CW 1995 Sexual conflict: males with highest mating success convey the lowest fertilization benefits to females. Proc. R. Soc. Lond. B 262, 135–139 (doi:10.1098/rspb.1995.0187) 10.1098/rspb.1995.0187 8524908

[37] Lorch PD Bussiere LF Gwynne DT 2008 Quantifying the potential for sexual dimorphism using upper limits on Bateman gradients. Behaviour 145, 1–24 (doi:10.1163/156853908782687205)

[38] Rodriguez-Munoz R Bretman A Slate J Walling CA Tregenza T 2010 Natural and sexual selection in a wild insect population. Science 328, 1269–1272 (doi:10.1126/science.1188102) 20522773 10.1126/science.1188102

[39] Fritzsche K Arnqvist G 2013 Homage to Bateman: sex roles predict sex differences in sexual selection. Evolution 67, 1926–1936 (doi:10.1111/evo.12086) 23815650 10.1111/evo.12086

[40] Ketterson ED Parker PG Raouf SA Nolan V Ziegenfus C Chandler CR 1998 Relative importance of extra-pair fertilizations to male and female reproductive success in dark-eyed juncos. In Avian reproductive tactics: female and male perspectives (eds Parker PG Burley NT), pp. 81–101 Lawrence, KS: Allen Press

[41] Worden BD Parker PG 2001 Polyandry in grain beetles, *Tenebrio molitor*, leads to greater reproductive success: material or genetic benefits? Behav. Ecol. 12, 761–767 (doi:10.1093/beheco/12.6.761)

[42] Clutton-Brock T 2007 Sexual selection in males and females. Science 318, 1882–1885 (doi:10.1126/science.1133311) 18096798 10.1126/science.1133311

[43] Clutton-Brock T 2009 Sexual selection in females. Anim. Behav. 77, 3–11 (doi:10.1016/j.anbehav.2008.08.026)

[44] Wedell N Karlsson B 2003 Paternal investment directly affects female reproductive effort in an insect. Proc. R. Soc. Lond. B 270, 2065–2071 (doi:10.1098/rspb.2003.2479) 10.1098/rspb.2003.2479 PMC1691472 14561296

[45] Alonzo SH Pizzari T 2010 Male fecundity stimulation: conflict and cooperation within and between the sexes. Am. Nat. 175, 174–185 (doi:10.1086/649596) 20028216 10.1086/649596

[46] Parker GA 1992 Evolutionary biology: snakes and female sexuality. Nature 355, 395–396 (doi:10.1038/355395a0)

[47] Gerlach NM McGlothlin JW Parker PG Ketterson ED 2012 Reinterpreting Bateman gradients: multiple mating and selection in both sexes of a songbird species. Behav. Ecol. 23, 1078–1088 (doi:10.1093/beheco/ars077)

[48] García-Navas V Ferrer ES Bueno-Enciso J Barrientos R Sanz JJ Ortegoa J 2014 Extrapair paternity in Mediterranean blue tits: socioecological factors and the opportunity for sexual selection. Behav. Ecol. 25, 228–238 (doi:10.1093/beheco/art111)

[49] Collias NE Collias EC 1996 Social organization of a red junglefowl, *Gallus gallus*, population related to evolution theory. Anim. Behav. 51, 1337–1354 (doi:10.1006/anbe.1996.0137)

[50] Etches RJ 1996 Reproduction in poultry. Wallingford, UK: CAB International

[51] Worley K Gillingham M Jensen P Kennedy LJ Pizzari T Kaufman J Richardson DS 2008 Single locus typing of MHC class I and class IIB loci in a population of red jungle fowl. Immunogenetics 60, 233–247 (doi:10.1007/s00251-008-0288-0) 18389232 10.1007/s00251-008-0288-0

[52] Marshall TC Slate J Kruuk LEB Pemberton JM 1998 Statistical confidence for likelihood-based paternity inference in natural populations. Mol. Ecol. 7, 639–655 (doi:10.1046/j.1365-294x.1998.00374.x) 9633105 10.1046/j.1365-294x.1998.00374.x

[53] Wade MJ 1979 Sexual selection and variance in reproductive success. Am. Nat. 114, 742–764 (doi:10.1086/283520) 10.1086/424531 15459886

[54] Wade MJ Arnold SJ 1980 The intensity of sexual selection in relation to male sexual behaviour, female choice, and sperm precedence. Anim. Behav. 28, 446–461 (doi:10.1016/S0003-3472(80)80052-2)

[55] Moorad JA Wade MJ 2013 Selection gradients, the opportunity for selection, and the coefficient of determination. Am. Nat. 181, 291–300 (doi:10.1086/669158) 23448880 10.1086/669158 PMC3620722

[56] Davison AC Hinkley DV 1997 Bootstrap methods and their application. Cambridge, UK: Cambridge University Press

[57] R Core Team 2012 *R: a language and environment for statistical computing*. Vienna, Austria: R Foundation for Statistical Computing. See http://www.R-project.org/

[58] Bolker BM Brooks ME Clark CJ Geange SW Poulsen JR Stevens MHH White JSS 2009 Generalized linear mixed models: a practical guide for ecology and evolution. Trends Ecol. Evol. 24, 127–135 (doi:10.1016/j.tree.2008.10.008) 19185386 10.1016/j.tree.2008.10.008

[59] Crawley MJ 2007 The R book. Hoboken, NJ: Wiley

[60] Pizzari T Cornwallis CK Løvlie H Jakobsson S Birkhead TR 2003 Sophisticated sperm allocation in male fowl. Nature 426, 70–74 (doi:10.1038/nature02004) 14603319 10.1038/nature02004

[61] Gowaty PA Hubbell SP 2009 Reproductive decisions under ecological constraints: it's about time. Proc. Natl Acad. Sci. USA 106, 10 017–10 024 (doi:10.1073/pnas.0901130106) 19528648 10.1073/pnas.0901130106 PMC2702798

[62] Kokko H Klug H Jennions MD 2012 Unifying cornerstones of sexual selection: operational sex ratio, Bateman gradient and the scope for competitive investment. Ecol. Lett. 15, 1340–1351 (doi:10.1111/j.1461-0248.2012.01859.x) 22925080 10.1111/j.1461-0248.2012.01859.x

[63] Schärer L Rowe L Arnqvist G 2012 Anisogamy, chance and the evolution of sex roles. Trends Ecol. Evol. 27, 260–264 (doi:10.1016/j.tree.2011.12.006) 22277154 10.1016/j.tree.2011.12.006

[64] Ah-King M 2013 On anisogamy and the evolution of ‘sex roles’. Trends Ecol. Evol. 28, 1–2 (doi:10.1016/j.tree.2012.04.004) 22964456 10.1016/j.tree.2012.04.004

[65] Neff BD Svensson EI 2013 Polyandry and alternative mating tactics. Phil. Trans. R. Soc. B 368, 20120045 (doi:10.1098/rstb.2012.0045) 23339236 10.1098/rstb.2012.0045 PMC3576579

[66] Froman DP Pizzari T Feltmann AJ Castillo-Juarez H Birkhead TR 2002 Sperm mobility: mechanisms of fertilizing efficiency, genetic variation and phenotypic relationship with male status in the domestic fowl, *Gallus gallus domesticus*. Proc. R. Soc. Lond. B 269, 607–612 (doi:10.1098/rspb.2001.1925) 10.1098/rspb.2001.1925 PMC1690926 11916477

[67] Cornwallis CK Birkhead TR 2006 Social status and availability of females determine patterns of sperm allocation in the fowl. Evolution 60, 1486–1493 16929665

[68] Pizzari T Cornwallis CK Froman DP 2007 Social competitiveness associated with rapid fluctuations in sperm quality in male fowl. Proc. R. Soc. B 274, 853–860 (doi:10.1098/rspb.2006.0080) 10.1098/rspb.2006.0080 PMC2093967 17251117

[69] Pizzari T Worley K Burke T Froman D 2008 Sperm competition dynamics: ejaculate fertilising efficiency changes differentially with time. BMC Evol. Biol. 8, 332 (doi:10.1186/1471-2148-8-332) 19087292 10.1186/1471-2148-8-332 PMC2627843

[70] Webster MS Tarvin KA Tuttle EM Pruett-Jones S 2007 Promiscuity drives sexual selection in a socially monogamous bird. Evolution 61, 2205–2211 (doi:10.1111/j.1558-5646.2007.00208.x) 17725624 10.1111/j.1558-5646.2007.00208.x

[71] Sousa BF Westneat DF 2013 Variance in mating success does not produce strong sexual selection in a polygynous songbird. Behav. Ecol. 24, 1381–1389 (doi:10.1093/beheco/art077)

[72] Schlicht E Kempenaers B 2013 Effects of social and extra-pair mating on sexual selection in blue tits (*Cyanistes caeruleus*). Evolution 67, 1420–1434 23617918 10.1111/evo.12073

[73] Løvlie H Cornwallis CK Pizzari T 2005 Male mounting alone reduces female promiscuity in the fowl. Curr. Biol. 15, 1222–1227 (doi:10.1016/j.cub.2005.05.060) 16005296 10.1016/j.cub.2005.05.060

[74] Pizzari T Birkhead TR 2001 For whom does the hen cackle? The adaptive significance of post oviposition cackling. Anim. Behav. 61, 601–607 (doi:10.1006/anbe.2000.1620)

[75] Coltman DW Bancroft DR Robertson A Smith JA Clutton-brock TH Pemberton JM 1999 Male reproductive success in a promiscuous mammal: behavioural estimates compared with genetic paternity. Mol. Ecol. 8, 1199–1209 (doi:10.1046/j.1365-294x.1999.00683.x) 10447860 10.1046/j.1365-294x.1999.00683.x

